# C3-Epimer of 25-Hydroxyvitamin D3 as a Superior Marker for Predicting the Severity of Chronic Kidney Disease in Rheumatoid Arthritis Patients

**DOI:** 10.1155/2022/5268706

**Published:** 2022-04-22

**Authors:** Jie Tang, Binwu Ying, Yuwei Yang, Bei Xu, Lin Yu, Wenqiang Jiang, Shutao Pu

**Affiliations:** ^1^Department of Laboratory Medicine, West China Hospital, Sichuan University, Chengdu 610041, China; ^2^Department of Laboratory Medicine, Mianyang Central Hospital, School of Medicine, University of Electronic Science and Technology of China, Mianyang 621000, China

## Abstract

**Objective:**

25-hydroxyvitamin D3 (25[OH]D3) is involved in oxidative stress regulation by upregulating the expression of antioxidant genes except for mineral homeostasis physiological role. C3-epimer of 25(OH)D3 (C3-epi-25[OH]D3) is a form of vitamin D metabolite with low bioaffinity in vivo, but little is known about the relationship between C3-epi-25(OH)D3 and diseases. This study was aimed at investigating the relationship between C3-epi-25(OH)D3 and the severity of chronic kidney disease (CKD) in patients with rheumatoid arthritis (RA).

**Method:**

A total of 318 RA inpatients were enrolled and divided into mild (*n* = 247), moderate (*n* = 46), and severe (*n* = 25) CKD groups according to the CKD prognosis criterion of the Kidney Disease Improving Global Outcomes guidelines. Serum levels of 25(OH)D2, 25(OH)D3, C3-epi-25(OH)D3, and free 25(OH)D (F25[OH]D) were measured, and the value of 25(OH)D was calculated. The relationship and changing trend of the indexes based on CKD severity were analyzed.

**Results:**

The serum levels of 25(OH)D, 25(OH)D3, and F25(OH)D showed a decreasing trend (*z* = −2.781–−3.996, *P* < 0.01) in RA patients with CKD progression from mild to severe, while C3-epi-25(OH)D3 showed an increasing trend (*z* = 6.741, *P* < 0.001) and 25(OH)D2 showed no significant difference among the groups (*z* = 0.976, *P* = 0.329). Only levels of C3-epi-25(OH)D3 presented significant differences between adjacent CKD severity groups in RA patients (mild to moderate: *z* = 3.963, *Padj* < 0.001; moderate to severe: *z* = 3.269, *Padj* = 0.002). Multiple logistic regression analysis showed that 25(OH)D3 and C3-epi-25(OH)D3 were significant predictors for CKD progression in RA patients, and C3-epi-25(OH)D3 had a better predictive advantage (moderate vs. mild: OR = 4.79, *P* < 0.001; severe vs. mild: OR = 17.85, *P* < 0.001).

**Conclusions:**

To the best of our knowledge, this is the first study to reveal that C3-epi-25(OH)D3 is a dominant predictor of CKD severity in RA patients. Further studies are needed to explore the relationship between C3-epi-25(OH)D3 and other diseases.

## 1. Introduction

Rheumatoid arthritis (RA) is a chronic systemic connective tissue inflammatory disease primarily characterized by arthritis and articular structure damage. As the disease progresses, chronic nonspecific connective tissue inflammation may spread to the entire body and involve major organs, such as the lungs, cardiovascular system, and kidneys, resulting in high disability and mortality [1]. Previous literature has described a large number of RA patients with hematuria, proteinuria, and renal dysfunction, and the incidence of chronic kidney disease (CKD) in RA patients is as high as 30% [1–3]. Meanwhile, some studies have reported CKD coexistence as the primary cause of death in RA patients, indicating that CKD is an independent risk factor for RA patient mortality [4–6].

Many studies have found that vitamin D (VitD) has anti-inflammatory, antioxidant, and immune regulatory characteristics and is associated with various diseases, including infections, tumors, kidney diseases, and autoimmune diseases [7, 8]. It has been confirmed that VitD deficiency or insufficiency exists in RA patients and is involved in RA development and prognosis [9, 10]. VitD is a fat-soluble prohormone, mainly including VitD2 and VitD3 forms, and can transform into more than 50 metabolites in vivo [11]. Among the metabolites, 25-hydroxyvitamin D (25[OH]D) and 1,25-dihydroxyvitamin D (1,25[OH]2D), which are formed by a series of hydroxylation, are essential for life [12]. They play important roles in regulating calcium and phosphorus metabolism and maintaining muscle, nerve, and cell functions [13]. Unlike 1,25(OH)2D, which is unstable and difficult to measure, 25(OH)D has the highest level, more stable form, and longer half-life in circulation and is recommended as the best marker for VitD nutritional evaluation by the Endocrine Society [14]. However, with the expansion and deepening of relevant research, some studies have found that 25(OH)D cannot accurately assess VitD nutritional status under some states, such as pregnancy, nephropathy syndrome, end-stage renal disease, coronary artery disease, and hemodialysis [15–19]. This may be attributed to the various 25(OH)D metabolites with different contents and bioaffinity. Meanwhile, some studies suggested that the presence of free 25(OH)D (F25[OH]D), a free form of 25(OH)D that cannot be measured by conventional methods, may also be a factor [15, 18]. In the past, limited by detection techniques, considerable studies about vitamin D focused on the total 25(OH)D concentrations without elucidating its subcomponents. Currently, ultrahigh performance liquid chromatography-tandem mass spectrometry (UHPLC-MS/MS), which can achieve the simultaneous measurement of 25(OH)D2, 25(OH)D3, and C3-epimer of 25(OH)D3 (C3-epi-25[OH]D3), is recommended as the “gold standard” method for the detection of 25(OH)D and its metabolites [20].

C3-epi-25(OH)D3 is formed from 25(OH)D3 through 3*β* to 3*α* isomerization transformation under the action of the C3-epimerase enzyme [21]. Its presence remains a troublesome issue in VitD nutritional assessment, because except for spatial conformation, C3-epi-25(OH)D3 has the same molecular weight and structural formula as 25(OH)D3 but has no biological activity [22]. This means that at the same level of total 25(OH)D3, a high level of C3-epi-25(OH)D3 is essentially equivalent to a reduction in 25(OH)D3 levels, which affects the amount of bioactive vitamin D conversion and results in VitD storage overestimation. Thus far, different concentrations of C3-epi-25(OH)D3 have been found in infants, children, pregnant women, healthy adults, and people with various diseases (e.g., autoimmune thyroid disease, RA, type 1 diabetes mellitus, and Alzheimer's) [23–25]. Most of these studies focused on the serum concentration levels of C3-epi-25(OH)D3 and its effect on VitD storage estimation but paid little attention to its relationship with disease occurrence and progression. Here, we focus on the relationship between F25(OH)D, 25(OH)D metabolites, especially C3-epi-25(OH)D3, and CKD severity in RA patients to provide laboratory support for risk identification, prevention, and control of RA coexisting with CKD.

## 2. Materials and Methods

### 2.1. Ethical Review

This was a case-control study conducted in the Department of Rheumatology of Mianyang Central Hospital, School of Medicine, University of Electronic Science and Technology in China. It was approved by the Ethics Committee of Mianyang Central Hospital (approval No. S2018085). All subjects signed an informed consent form.

### 2.2. Patients and Grouping

A total of 318 RA patients were enrolled at Mianyang Central Hospital from January 2018 to October 2021, including 68 males and 250 females, with ages ranging from 27 to 77 years old (average: 57.7 ± 11.4). The inclusion criteria were as follows: (1) RA patients were diagnosed by physicians in the rheumatology department according to the American College of Rheumatology (ACR) criteria [26]; and (2) patients with an RA history of more than five years. The exclusion criteria were as follows: (1) patients who had undergone kidney transplantation or dialysis; (2) patients with thyroid dysfunction, malignant tumors, coinfection, hypertension, diabetes, or other immune system diseases; (3) pregnant patients; and (4) patients receiving VitD supplement, calcium, or hormones within one month of the study. Next, according to the CKD prognosis criterion of the Kidney Disease Improving Global Outcomes 2012 guideline [27], and based on estimated glomerular filtration rate (eGFR) and urine albumin-to-creatinine ratio (ACR) measured every 1–2 months for the last 3–6 months in the medical records, all enrolled RA patients were divided into three groups with different severities of CKD: (1) mild CKD group (247 cases): eGFR ≥ 60 ml/min/1.73m^2^ and ACR < 30 mg/g; (2) moderate CKD group (46 cases): eGFR ≥ 60 ml/min/1.73m^2^ and 30 mg/g < ACR ≤ 300 mg/g*, or*45 ml/min/1.73m^2^ ≤ eGFR < 60 ml/min/1.73m^2^ and ACR < 30 mg/g; and (3) severe CKD group (25 cases): eGFR ≥ 60 ml/min/1.73m^2^ and ACR ≥ 300 mg/g*, or*45 ml/min/1.73m^2^ ≤ eGFR < 60 ml/min/1.73m^2^ and ACR ≥ 30 mg/g*, or*eGFR < 45 ml/min/1.73m^2^.

### 2.3. Sample Collection

Blood samples were collected after obtaining informed consent from all patients. Approximately 3 ml fasting blood from every subject was collected in an SST-II vacuum tube (BD, USA), and serum was collected after centrifugation at 4000 rpm (approximately 1500 × *g*) for 10 min.

### 2.4. Laboratory Measurements

Jasper™ HPLC (Shimadzu, Japan)/AB SCIEX Triple Quad™ 4500MD (ABI, USA) UHPLC-MS/MS analyzer and lipid-soluble vitamin assay kits (Fandi Biotechnology Co. Ltd, Chengdu) were used to measure 25(OH)D2, 25(OH)D3, and C3-epi-25(OH)D3 levels; the sum of the three was the value of 25(OH)D. RT-6100 ELISA analyzer (Rayto Life and Analytical Sciences Co., Ltd., Shenzhen) and F25(OH)D ELISA kit (DIA Source Future Diagnostics, Belgium) were used to measure F25(OH)D levels. Cobas c701 automatic biochemical analyzer (Roche, USA) and original reagents were used to measure high-sensitivity C-reactive protein (hsCRP) levels. BN II automatic specific protein analyzer (Siemens, DE) and original reagents were used to measure rheumatoid factor (RF) levels. Measurements were performed following calibration and quality control.

### 2.5. Isolation and Identification of 25(OH)D

Serum samples of 200 *μ*l were mixed with 10 *μ*l internal standard (a mixture of 25[OH]D2-d6, 25[OH]D3-d6, and C3-epi-25[OH]D3-[^2^H3]). Samples were then vortex mixed with 1.0 ml tert-butyl methyl ether release agent (CNW, Germany) for 5 min and centrifuged at 13,000 rpm (12000 × *g*) for 5 min. We collected 800 *μ*l of supernatant and dried this using an MD200-1A Nitrogen Evaporator (ALLSHENG Instrument Co., Ltd., Hangzhou, China). Next, we redissolved the sample with 100 *μ*l methanol aqueous solution containing 0.1% formic acid, mixed for 2 min, and then centrifuged at 13,000 rpm for 5 min. Finally, we collected the supernatant to measure 25(OH)D2, 25(OH)D3, and C3-epi-25(OH)D3 levels using the Jasper™ HPLC (Shimadzu, Japan)/AB SCIEX Triple Quad™ 4500MD (ABI, USA) UHPLC-MS/MS analyzer.

The HPLC conditions were as follows: F5 column at 40°C, 0.6 ml/min flow rate, 10 *μ*l injection volume, and density gradient elution by mobile phase A of 0.1% formic acid in deionized water and mobile phase B of 0.1% formic acid in methanol.

The MS conditions were as follows: curtain gas (CUR) 25 psi, atomized gas (GS1) 40 psi, heated gas (GS2) 30 psi, collision gas (CAD) 6 psi, solvent removal temperature (TEM) 350°C, impact chamber injection voltage (CXP) 6 V, declustering potential (DP) 130 V, and collision voltage (CE) 16 V. Mass spectrometry analysis used the atmospheric pressure chemical ionization (APCI) ion source and a positive ion multiple reaction monitoring mode with a residence time of 40 ms. Chromatograms and process data were analyzed using Analyst® MD software (version number: 1.6.3) and Multiquant™ MD software (version number: 3.0.2).

### 2.6. Statistical Analysis

Statistical analyses were performed using MedCalc 18.2 (MedCalc Software, Mariakerke, Belgium) or SPSS 19.0 (SPSS Inc., Chicago, IL, USA). After normality testing with the one-sample Kolmogorov–Smirnov method, the normal distribution measurement data were presented as the mean ± standard deviation. Difference analysis among multiple groups was performed using one-way ANOVA. The comparison between any two groups was performed using LSD tests, and the significance level was corrected as *α*/*k* (*k* was the number of comparisons) using the Bonferroni method. The nonnormal measurement data were presented as *M* (*P*_25_, *P*_75_). Difference analysis among multiple groups was performed using the independent sample Kruskal–Wallis test. The comparison between any two groups was performed using post hoc tests of Kruskal–Wallis, and the adjusted *P* value (*Padj*) was used to determine whether there were significant differences. The trend of 25(OH)D metabolites with the severity of CKD was analyzed using the independent sample Jonckheere–Terpstra test. The significance of the change degree in 25(OH)D metabolites for the two groups was analyzed using post hoc tests of Jonckheere–Terpstra, and the *Padj* was used to determine the significance between the two groups. The association between the severity of CKD and the serum levels of 25(OH)D metabolites in RA patients was analyzed using multiple logistic regression. Statistical significance was set at *P* < 0.05 (if multiple comparison or multiple logistic regression), *Padj* < 0.05 (if post hoc test), or *P* < 0.017 (if Bonferroni correction).

## 3. Results

### 3.1. Isolation and Identification of C3-Epi-25(OH)D3

**Figure 1:**
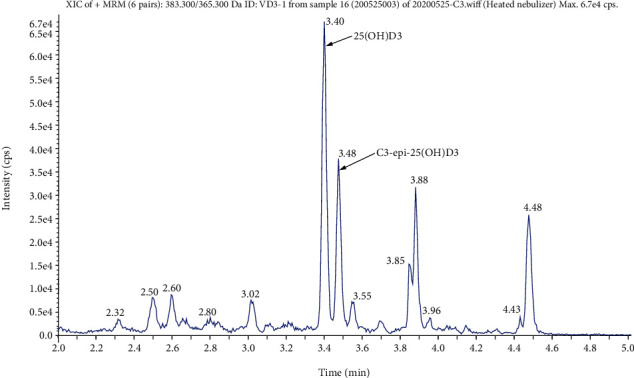
Different retention times of 25(OH)D3 and C3-epi-25(OH)D3. Note: 25(OH)D3 and C3-epi-25(OH)D3 have identical monoisotopic masses and fragmentation patterns (Q1/Q3 mass = 383.3 Da/365.3 Da) but were separated according to different retention times (25[OH]D3 = 3.40 min; C3 − epi − 25[OH]D3 = 3.48 min).

The identification figure of C3-epi-25(OH)D3 was obtained by monitoring the Q1/Q3 mass = 383.3 Da/365.3 Da ion pair (Figure 1). The results showed that the retention time of 25(OH)D3 was 3.40 min, and that of C3-epi-25(OH)D3 was 3.48 min.

### 3.2. General Information of RA Patients with Different CKD Severities

The basic clinic information and laboratory data of different CKD severity groups of RA patients are shown in Table 1. There were no significant differences in age (*F* = 2.303, *P* = 0.102) and gender (*χ*^2^ = 0.575, *P* = 0.750) among the three groups. All indexes had significant differences among the three groups (*χ*^2^ = 8.792–165.496, *P* < 0.05), except for 25(OH)D2 (*χ*^2^ = 1.012, *P* = 0.603) and RF (*χ*^2^ = 3.033, *P* = 0.220).

Multiple comparisons for 25(OH)D metabolites showed that C3-epi-25(OH)D3 levels in the severe CKD group > moderate CKD group > mild CKD group (severe vs. moderate: *z* = 2.726, *Padj* = 0.019; severe vs. mild: *z* = 6.193, *Padj* < 0.001; moderate vs. mild: *z* = 3.876, *Padj* < 0.001). Levels of 25(OH)D (*z* = −2.821, *Padj* = 0.014) and F25(OH)D (*z* = −2.731, *Padj* = 0.019) in the severe CKD group were lower than those in the mild CKD group. Levels of 25(OH)D3 in the moderate CKD group (*z* = −2.702, *Padj* = 0.021) and severe CKD group (*z* = −3.238, *Padj* = 0.004) were lower than those in the mild CKD group. Other multiple comparisons had no significant differences (*z* = 0.989–1.475, *Padj* > 0.05).

### 3.3. The Trend of 25(OH)D Metabolites in Different CKD Severities

**Figure 2:**
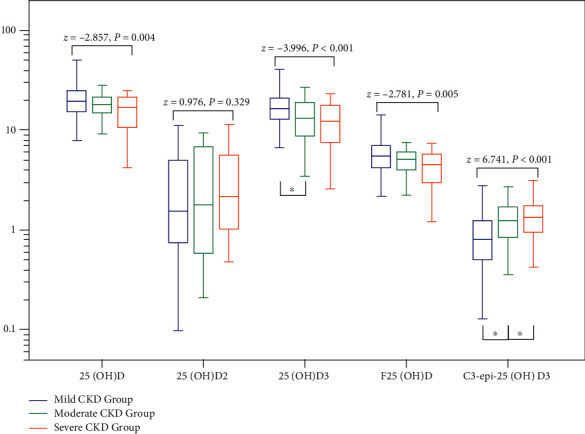
Trend of 25(OH)D metabolites in RA patients with the severity of CKD (Unit: ng/ml). Note: ^∗^*Padj* < 0.05. Among the observed 25(OH)D metabolites, only changes in C3-epi-25(OH)D3 levels were significantly different between adjacent CKD severity groups (*Padj* < 0.05). 25(OH)D3 levels were significantly different only from the mild to moderate CKD severity group, and there was no significant difference in the levels of 25(OH)D, 25(OH)D2, and F25(OH)D.

According to the ordered Jonckheere–Terpstra test (Figure 2), the levels of 25(OH)D, 25(OH)D3, and F25(OH)D in RA patients showed a significant decreasing trend with the deterioration of CKD (*z* = −2.781–−3.996, *P* < 0.01). On the contrary, the level of C3-epi-25(OH)D3 presented a significant increasing trend (*z* = 6.741, *P* < 0.001). Levels of 25(OH)D2 showed no significant trend (*z* = 0.976, *P* = 0.329). Multiple comparisons showed that C3-epi-25(OH)D3 levels changed significantly from mild to moderate CKD (*z* = 3.963, *Padj* < 0.001) and from moderate to severe CKD (*z* = 3.269, *Padj* = 0.002), whereas 25(OH)D3 levels changed significantly only from mild to moderate CKD (*z* = −2.755, *Padj* = 0.009).

### 3.4. Value of Risk Prediction of 25(OH)D Metabolites to CKD Severity in RA Patients

Because 25(OH)D and F25(OH)D had multicollinearity with 25(OH)D2 and 25(OH)D3 data, they were excluded from our multiple logistic regression analysis. Compared to mild CKD, 25(OH)D3 was a protective factor for moderate CKD (OR = 0.86, *P* < 0.001) and severe CKD (OR = 0.72, *P* < 0.001). C3-epi-25(OH)D3 was a risk factor for moderate CKD (OR = 4.79, *P* < 0.001) and severe CKD (OR = 17.85, *P* < 0.001). 25(OH)D2 was not a factor for moderate and severe CKD (*P* > 0.05). After adjusting for sex, age, hsCRP, and RF, the results were consistent with those before adjustment (Table 2).

## 4. Discussion

Our study firstly reports C3-epi-25(OH)D3 as a potential biomarker for predicting the severity of CKD in RA patients. Previous epidemiological studies suggested that the complexity of VitD metabolites and ethnic and regional differences were the reasons for the uncertain relationship between RA severity and VitD levels and did not consider C3-epi-25(OH)D3 due to technical limitations [28]. C3-epi-25(OH)D3 was first found in infants in 2006, attributed to the development of high sensitivity HPLC-MS/MS technology [29]. It was then proposed to be measured at the National Health and Nutrition Examination Surveys (NHANES) Roundtable in 2007 [30]. Subsequently, the first report of 2007–2010 NHANES regarding the nationally representative 25(OH)D determination showed that the rate of C3-epi-25(OH)D3 in the American population was as high as 86% [31]. From then on, increasing studies have focused on the relationship between C3-epi-25(OH)D and disease or health [24, 32, 33]. However, most of these have been limited to the VitD storage overestimation, ignoring the role of C3-epi-25(OH)D in disease progression [34, 35], and thus failing to provide a more reasonable explanation for the abnormal increase of C3-epi-25(OH)D in pathological conditions. Therefore, this study focused on the difference in C3-epi-25(OH)D levels in RA patients with different severities of CKD and aimed to understand the potential connections between the two.

We investigated the serum levels of primary 25(OH)D metabolites, including 25(OH)D2, 25(OH)D3, C3-epi-25(OH)D3, and F25(OH)D, in RA patients with different CKD severities and measured the changing trend with the progression of CKD. With CKD deterioration, the levels of C3-epi-25(OH)D3 showed an overall significantly increasing trend in all RA patients. C3-epi-25(OH)D3 levels increased significantly from mild to moderate CKD, as well as from moderate to severe CKD. On the contrary, the levels of 25(OH)D, 25(OH)D3, and F25(OH)D showed an overall downward trend with CKD deterioration. However, when comparing adjacent groups, 25(OH)D3 levels significantly declined only from mild to moderate CKD. Our results suggest that 25(OH)D3 and C3-epi-25(OH)D3 may be potential markers for CKD progression in RA patients.

Subsequently, we analyzed the association between these metabolites and the risk of CKD severity in RA patients before and after adjusting for sex, age, inflammatory factor (hsCRP) levels, and specific antibody (RF) titers. The results confirmed that both 25(OH)D3 and C3-epi-25(OH)D3 were predicting factors for CKD severity in RA patients. With the deterioration of CKD in RA patients, 25(OH)D3 levels had a progressive negative association, while C3-epi-25(OH)D3 levels had a more significant progressive positive association. These results indicate that C3-epi-25(OH)D3 is a reasonable marker for predicting CKD in RA patients.

Based on these findings, we report that C3-epi-25(OH)D3 is an applicable predictor of CKD severity in RA patients; however, further studies are required to verify this in other diseases. To the best of our knowledge, few studies have focused on the role of C3-epi-25(OH)D3 in disease progression. Previous studies have indirectly mentioned a possible role of C3-epi-25(OH)D3 in disease progression, though their conclusions have been reported as inconclusive. Recent research has showed that although no association was found using Mendelian randomization, C3-epi-25(OH)D3 levels were positively associated with diabetes mellitus occurrence and progression in a meta-analysis [36]. Two other studies about the genetic model of C3-epi-25(OH)D3 variation found that its genetic determinants and potential factors were different from 25(OH)D3 [37, 38]. Increased C3-epi-25(OH)D3 levels were strongly correlated to 25(OH)D3 levels in the general population, but in individuals with diabetes mellitus, this correlation was significantly weakened [38]. These results suggested that the physiological and pathological significance of C3-epi-25(OH)D3 cannot follow that of 25(OH)D3. The abnormal increase in C3-epi-25(OH)D3 in disease states may have other causes, which remain to be elucidated. Thus, further exploration about its clinical relevance, etiology, and potential effect concerning VitD status is needed.

This study also considered and excluded the effect of exogenous intake of VitD supplements on the results. Two studies have analyzed the effect of oral supplementation or overingestion of VitD on C3-epi-25(OH)D3 in canines [39] and mice [40] and found that it may cause increased levels of C3-epi-25(OH)D3 in vivo. However, experimental data in humans are currently lacking. In order to reduce the influencing factors and ensure the accuracy of the results, we excluded patients who took VitD supplements within one month of the study. However, there were still some limitations in our study. The number of patients with extremely severe CKD was so small that these patients could not be listed separately from the CKD severity group. Thus, the value of C3-epi-25(OH)D3 for RA patients with extremely severe CKD was not determined. In addition, other potentially influential factors, such as sunlight exposure, diet habits, and use of sunscreen, were not considered. Lastly, C3-epi-25(OH)D2 was not included in this study because its levels were too low to be detected by our UHPLC-MS/MS technique.

## 5. Conclusions

In conclusion, we report the potential predictive value of C3-epi-25(OH)D3 for CKD progression risk in RA patients. However, this was a cross-sectional study of a single institution, and our conclusions need to be verified using multicenter and larger samples. We must also determine whether this conclusion can be applied to CKD progression caused by other diseases, and whether C3-epi-25(OH)D3 takes part in the occurrence and development of other diseases and is valid for diagnosis, treatment, and prognosis. Further research and discussion are needed to acquire a better understanding of C3-epi-25(OH)D3 and its relationship with disease mechanisms.

## Figures and Tables

**Table 1:** Comparison of observed indicators in RA patients with different CKD severity groups.

Indicator	Mild CKD (*n* = 247)	Moderate CKD (*n* = 46)	Severe CKD (*n* = 25)	*F*/*χ*^2^, *P*
Sex (male/female)^▲^	53/194	17/29	8/17	5.850, 0.054
Age (years)	56.2 ± 10.9	62.9 ± 10.9	63.8 ± 11.0	2.303, 0.102
25(OH)D (ng/ml)	19.55 (15.19, 24.84)	18.275 (14.83, 21.62)	16.72 (10.61, 21.54)^∗^	9.335, 0.009
25(OH)D2 (ng/ml)	1.55 (0.75, 4.96)	1.78 (0.58, 6.82)	2.17 (1.02, 5.59)	1.012, 0.603
25(OH)D3 (ng/ml)	16.36 (13.04, 20.97)	13.18 (8.81, 19.00)^∗^	12.22 (7.54, 17.61)^∗^	15.911, <0.001
F25(OH)D (ng/ml)	5.45 (4.20, 6.97)	5.13 (4.00, 6.10)	4.48 (2.98, 5.81)^∗^	8.792, 0.012
C3-epi-25(OH)D3 (ng/ml)	0.80 (0.50, 1.26)	1.24 (0.84, 1.72)^∗^	1.73 (1.34, 2.17)^∗^^#^	48.302, <0.001
Cr (mol/l)	51.9 (44.4,60.1)	64.8 (53.3,74.9)^∗^	93.5 (86.7,120.8)^∗^^#^	77.410, <0.001
CysC (mg/l)	0.97 (0.86,1.12)	1.42 (1.35,1.54)^∗^	1.92 (1.57,2.24)^∗^^#^	165.496, <0.001
eGFR (ml/min/1.73m^2^)	81 (70.1, 90.9)	55.8 (51.9, 58.1)∗	41.5 (37.0, 51.1)^∗^^#^	159.598, <0.001
ACR (mg/g)	5.19 (3.14,9.79)	25.47 (12.76,40.11)	31.49 (13.80,146.81)	42.284, <0.001
hsCRP (mg/l)	12.85 (3.38, 36.46)	23.53 (8.05, 56.23)^∗^	27.72 (9.93, 59.17)^∗^	26.269, <0.001
RF (IU/ml)	101.5 (25.4, 336.0)	77.0 (11.2, 300.5)	136.5 (12.2, 313.0)	3.033, 0.220

Note: ^▲^Crosstabs *χ*^2^ test of 2 × 3 tabulations was used, and the rest were performed by Kruskal-Wallis test (nonnormal distribution) or one-way AVONA analysis (normal or nearly normal distribution). ^∗^Severe or moderate *vs.* mild CKD group, *Padj* < 0.05; ^#^severe *vs.* moderate CKD group, *Padj* < 0.05. Among 25(OH)D metabolites, only C3-epi-25(OH)D3 levels had significant differences between any two groups of the three different CKD severity groups (all *Padj* < 0.05).

**Table 2:** Prediction value of 25(OH)D metabolites on the severity of CKD in RA patients.

CKD severity	Metabolites	Crudely analysis			Adjusted analysis^∗^		
		OR (95% CI)	*Wald* *χ*^2^	*P*	OR (95% CI)	*Wald* *χ*^2^	*P*
Moderate	25(OH)D2	1.12 (0.99, 1.25)	3.73	0.053	1.11 (0.99, 1.25)	3.214	0.073
	25(OH)D3	0.86 (0.81, 0.92)	19.163	<0.001	0.87 (0.81, 0.93)	17.456	<0.001
	C3-epi-25(OH)D3	4.97 (2.70, 9.14)	26.518	<0.001	4.79 (2.59, 8.83)	25.18	<0.001
Severe	25(OH)D2	1.08 (0.91, 1.29)	0.786	0.375	1.09 (0.91, 1.31)	0.844	0.358
	25(OH)D3	0.72 (0.64, 0.82)	26.449	<0.001	0.72 (0.63, 0.82)	25.68	<0.001
	C3-epi-25(OH)D3	17.77 (6.04, 52.27)	42.078	<0.001	17.85 (5.97, 53.32)	41.005	<0.001

Note: ^∗^Adjusted factors include age, sex, hsCRP, and RF. The results above were compared with the mild CKD severity. Of the 25(OH)D metabolites observed, C3-epi-25(OH)D had a better predictive value for both moderate CKD (OR = 4.97, adjusted OR = 4.79, *P* < 0.001) and mild CKD (OR = 17.77, adjusted OR = 17.85, *P* < 0.001).

## Data Availability

The datasets used and analyzed during the current study are available from the corresponding author on reasonable request.

## Abbreviations

1,25(OH)2D: 1,25-dihydroxyvitamin D
25(OH)D: 25-hydroxyvitamin D
C3-epi-25(OH)D: C3-epimer of 25-hydroxyvitamin D
ACR: American College of Rheumatology
CKD: Chronic kidney disease
eGFR: Estimated glomerular filtration rate
F25(OH)D: Free 25(OH)D
UHPLC-MS/MS: Ultrahigh high-performance liquid chromatography-tandem mass spectrometry
hsCRP: Hypersensitive C-reactive protein
KDIGO: Kidney disease: Improving global outcomes
RA: Rheumatoid arthritis
RF: Rheumatoid factor
uACR: Urinary albumin to creatinine ratio
VitD: Vitamin D.
